# The emerging role of Th1 cells in atherosclerosis and its implications for therapy

**DOI:** 10.3389/fimmu.2022.1079668

**Published:** 2023-01-05

**Authors:** Jiaojiao Chen, Xuying Xiang, Lei Nie, Xiaoqing Guo, Feng Zhang, Cheng Wen, Yuanpeng Xia, Ling Mao

**Affiliations:** Department of Neurology, Union Hospital, Tongji Medical College, Huazhong University of Science and Technology, Wuhan, China

**Keywords:** atherosclerosis, Th1 cells, inflammation, cytokines, therapy

## Abstract

Atherosclerosis is a chronic progressive inflammatory disease of the large and medium-sized artery walls. The molecular mechanisms regulating the onset and progression of atherosclerosis remain unclear. T cells, one of the most common immune cell types in atherosclerotic plaques, are increasingly recognized as a key mediator in the pathogenesis of atherosclerosis. Th1 cells are a subset of CD4^+^ T helper cells of the adaptive immune system, characterized by the expression of the transcription factor T-bet and secretion of cytokines such as IFN-γ. Converging evidence shows that Th1 cells play a key role in the onset and progression of atherosclerosis. Besides, Th1 is the central mediator to orchestrate the adaptive immune system. In this review, we aim to summarize the complex role of Th1 cells in atherosclerosis and propose novel preventative and therapeutic approaches targeting Th1 cell-associated specific cytokines and receptors to prevent atherogenesis.

## 1 Introduction

Atherosclerosis is among the most common causes of cardiovascular disease (CVD), including stroke, acute coronary syndrome, peripheral artery disease, and sudden death (1). Although our understanding of atherosclerosis has made significant progress during the past several decades, its etiology and pathogenesis remain unclear. Atherosclerosis is triggered by the low-density lipoprotein (LDL) retained in the large and medium artery walls (2), which exerts a more pro-atherosclerotic effect when modified to form oxidized low-density lipoprotein (ox-LDL) (2). Although the retention of lipids is considered the initial essential step, considerable evidence suggests that it is insufficient to trigger and maintain atherosclerosis development. It is believed that atherosclerosis is a chronic immune-inflammatory disease (3). The immune system is indispensable in the induction, progression, and plaque disruption (plaque rupture or plaque erosion) of plaques (4). Besides, evidence has suggested that activated T cells exist in human atherosclerotic plaques (5). T cells, since first identified in human atherosclerotic plaques thirty years ago, are increasingly believed as a key mediator in the pathogenesis of atherosclerosis (6). Single-cell level tests in mouse and human aortic plaques, such as single-cell RNA-sequencing and cytometry by time of flight (CyTOF), found that 25%-38% of the cells in plaques were T cells (7). The predominant T cell subtype in plaques are T helper 1 (Th1) cells, a subgroup of CD4**^+^** T cells. Th1 cells are believed to be the most pro-atherogenic subset capable of accelerating inflammation, plaque instability, and lesion growth (8, 9). In ApoE^-/-^ mice, atherosclerosis was reduced without T-bet, IFN-γ (Th1 cells characteristic cytokine), or IFN-γ receptors (10). In contrast, regulatory T (Treg) cells have anti-atherogenic roles. However, some studies have revealed that Treg’s plasticity makes it possible to become pro-atherogenic in advanced atherosclerosis (7, 11). Whereas the role of other Th cells, such as Th2, Th9, and Th17, seems to be more complex and less understood in the development of atherosclerosis (7). However, the specific pro-atherogenic mechanism of Th1 cells has not been fully elucidated. In addition, most of the mechanisms have been obtained in experimental models and are not fully representative of the human immune microenvironment (12). Translating these findings to human-related atherosclerotic disease remains a considerable challenge. The primary purpose of this review is to summarize and update the current knowledge associated with the role of Th1 in atherosclerosis.

Understanding the role of Th1 cells in atherosclerosis may lead to identifying therapeutic targets for treatment. This review aims to summarize the role of Th1 cells in atherosclerosis and the recent progression related to Th1 cell research in recent years. Although multiple therapies modulate atherosclerosis, the strategies mainly focus on reducing atherosclerosis-associated risk factors and cholesterol or triglyceride metabolism modulation to alleviate hyperlipidemia. In this review, we will focus on the role of Th1 cells in atherosclerosis development. Besides, it is essential to provide novel Th1 cell-associated therapy methods to prevent cardiovascular events driven by chronic inflammation.

## 2 Th1 cells formation and activation

### 2.1 Development of Th0 cells

**Figure 1:**
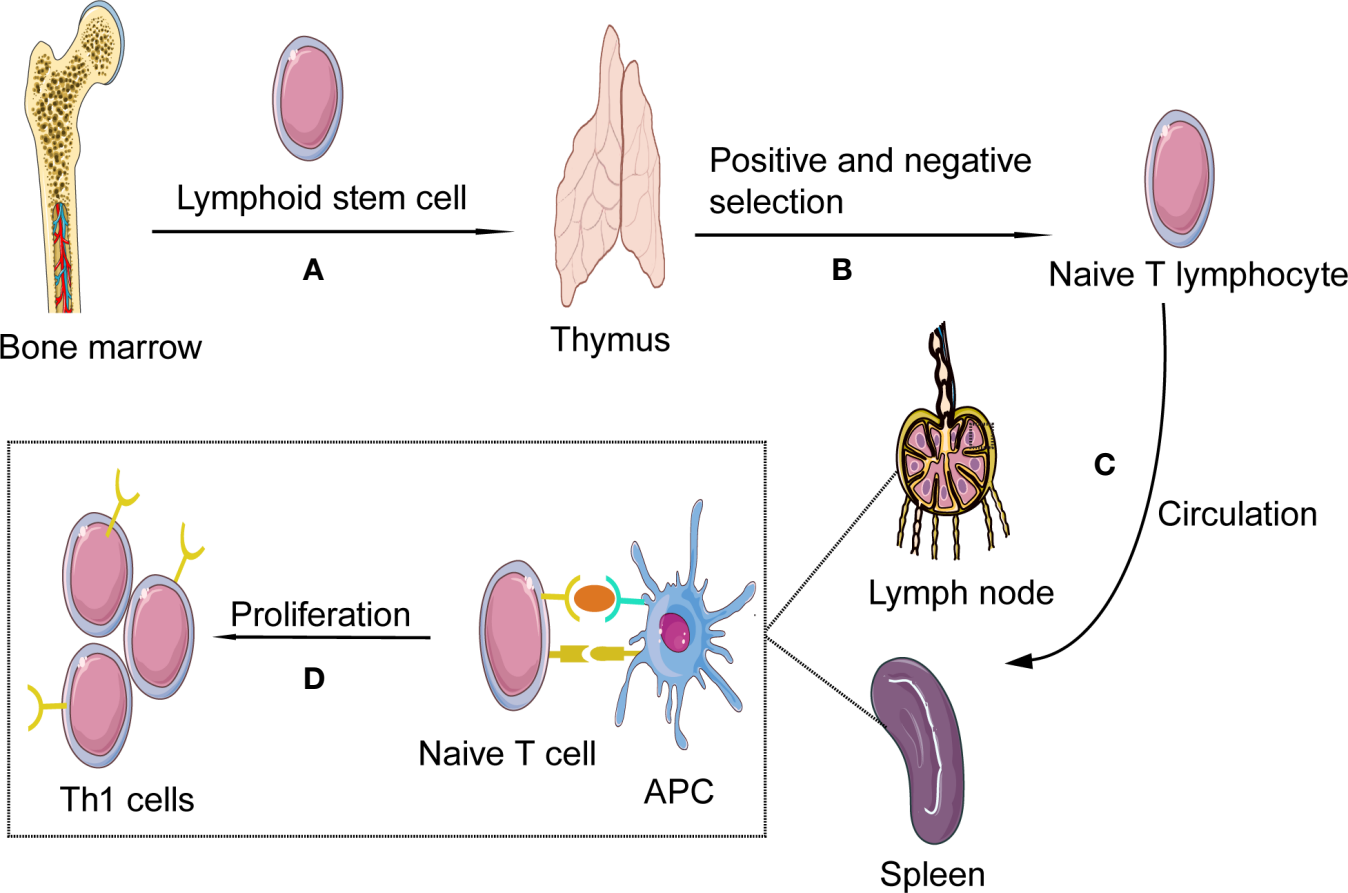
Th1 cells formation and activation. **(A)** Th1 cells, deriving from hematopoietic progenitor cells in bone marrow, become lymphoid stem cells and migrate to the thymus where they expand as thymocytes through blood circulation; **(B)** In the process of thymus development and maturation, the thymocytes undergo several steps of positive and negative selection. **(C)** Finally naïve T cells are released from the thymus into the circulation; In the blood and lymphoid circulation, the chances are high for them to detect presented antigens on antigen presenting cells (APCs), such as ox-LDL and HSP 60/65. **(D)** As the naïve T cell encounters its antigen the local milieu will prime the T cell to shift into Th1; The naïve T cell will then start to produce cytokines and proliferate, developing into effector cells and memory cells.

Th1 cells, as one subtype of CD4+ cells, are differentiated from Th0 cells. Th0 cells, also known as naïve CD4+ T cells, develop in the thymus, providing a specific microenvironment essential for T lymphocyte differentiation and have not yet experienced the stimulation of antigens. In tissue and circulation, due to the number of α/β+T cells is much larger than that of γ/δ+ T cells, so we mainly discuss α/β+T cells in this review (13). Th0 cells develop in the thymus, where bone-marrow-derived lymphoid progenitor cells gradually develop into double negative pro-T cells(CD4-CD8-). This stage involves activating recombinase genes 1 and -2 to rearrange the TCR genes and generate a random, unique TCR on each T cell. Furthermore, at this stage, T cells divide into either α/β+ or γ/δ+ T cells. Then under IL-7stimulation, CD4 and CD8 expression levels on the cell surface is up-regulated, and α/β+ T cells develop into double-positive (CD4+CD8+) pre-T cells. At the same time, the cells stop proliferating and begin to rearrange α genes, which, together with β chain genes form mature TCR, suggesting the formation of immature T cells and, after positive selection, eventually develop into naive CD4+CD8− T-helper cells. After negative selection, naive CD4+ T cells (Th0 cells) were obtained. In the negative selection process, most self-reactive T cells are eliminated by induced apoptosis, but this process does not eliminate all autoreactive CD4+ T cells (14–17). Afterward, Th0 cells leave the thymus and migrate to the secondary lymphatic organs(such as lymph nodes and spleen) (18) (**Figure 1**).

### 2.2 Transformation from Th0 to Th1 cells

Experimental evidence has shown that the Th1 cell surface markers in the plaques are characterized by a high expression level of CD44, a T cell activation marker. In contrast, the L-selectin expression level is low, suggesting that they are not naïve T cells (7, 19). Simultaneously, TCR sequencing revealed that these cells were oligoclonal expansion (20), suggesting that Th1 cells are mainly activated by specific antigens and proliferate in plaques. Following activation by antigen-presenting cells in second lymphoid organs, naive CD4+ T cells differentiate into central memory or effector memory T-cells (21). Central memory T-cells generally reside in lymphoid tissues and are primed for rapid response to a previously encountered antigen. After activation, central memory T-cells undergo clonal expansion and differentiate into effector memory T-cells. Some studies have revealed that the activation and proliferation of Th1 cells happen in the aorta (19).

#### 2.2.1 Activation of Th0 cells by antigens

Ox-LDL, its core apolipoprotein ApoB100, and the heat shock protein 60/65 (HSP60/65) produced by stressed vascular endothelial cells may represent self-antigens that can induce atherosclerosis (22–24). Single-cell sequencing revealed that ApoB reactive T cells have different helper T cell phenotypes, thus playing different roles in atherosclerosis. However, their role in atherosclerosis is still unclear due to the limitations of research methods and tools (25).

Furthermore, T cells in plaques have epitope-specific T cell receptors (TCRs) for influenza virus, coronavirus, and others (26). These TCRs share the same antigenic epitopes as associated protein sequences found on endothelial and smooth muscle cells, suggesting the possibility of autoimmune-mediated T cell activation in the absence of an active infection. These findings suggest that T cells located in plaques might have a potential response to self-epitopes by monoclonal expansion under self-antigens participation (26).

Th0 cells do not recognize antigens directly. Th0 cells activation is associated with professional antigen-presenting cells (APCs) such as B lymphocytes, macrophages, and several subsets of dendritic cells that present peptide antigens through the major histocompatibility complex class II (MHCII) (27). Recent evidence indicates that specific pattern recognition receptors (such as toll-like receptors) expressed on innate immune cells’ surfaces, are also expressed on T lymphocytes during infection or inflammation, which can provide co-stimulatory signals to induce Th0 cells activation and differentiation (28). Naïve T cell activation needs the help of antigen peptide-MHCII complex, and the co-stimulatory molecules expressed on the APCs surface, leading to clonal expansion and effector T cell differentiation. Otherwise, the T cells will not be fully activated and undergo apoptosis or anergy (27). TCR interacts with MHCII molecular complex to deliver activating signals. In ApoE^-/-^ mice, lacking MHC II, which is required to activate Th0 cells, are characterized by decreased severity of atherosclerosis. However, a recent study has discovered that although the level of CD4^+^ T cells and inflammatory cytokines are lower in ApoE^-/-^/MHCII^-/-^ mice, their atherosclerotic lesions seem significantly more severe than control groups (10). This result indicates that the antigen peptide-MHCII molecular complex plays an indispensable role in inducing the activation of Treg cells, considered essential for their athero-protective action. Cell transplantation and specific antibody-blocking experiments suggest that the loss of Treg cells may be responsible for the exacerbation of atherosclerosis in MHCII-deficient mice (10). These experimental pieces of evidence suggest that MHCII molecules present on the surface of different B cells exert opposite effects by interacting with different target T cells.

#### 2.2.2 Full activation of Th0 cells with the help of co-stimulatory molecules

Many co-stimulatory such as CD28-B7(CD80/CD86) and CD40-CD40L are involved in Th1 cells activation. The best-defined and well-studied co-stimulatory molecule in the context of atherosclerosis is the B7 family (29). The B7 family members B7-1 (CD80) and B7-2 (CD86) are mainly on the professional antigen-presenting cells’ surface. Both bind to CD28, a homologous dimer protein belonging to the immunoglobulin receptor superfamily constitutively expressed on almost all mature murine T cells (30). CD28-B7(CD80/CD86), together with the TCR signaling pathway, promotes IL-2 gene expression, cellular proliferation, and cytokine secretion. Interleukin-2 (IL-2), acting in an autocrine fashion, can sustain T cell proliferation (31). It has been suggested that the CD28 co-stimulation pathway mainly promotes Th2 cell differentiation (32). However, the experiment conducted on mouse cells *in vivo* showed that CD28 co-stimulation is a crucial driver of IFN-γ secretion in Th1 cells (33).

**Figure 2:**
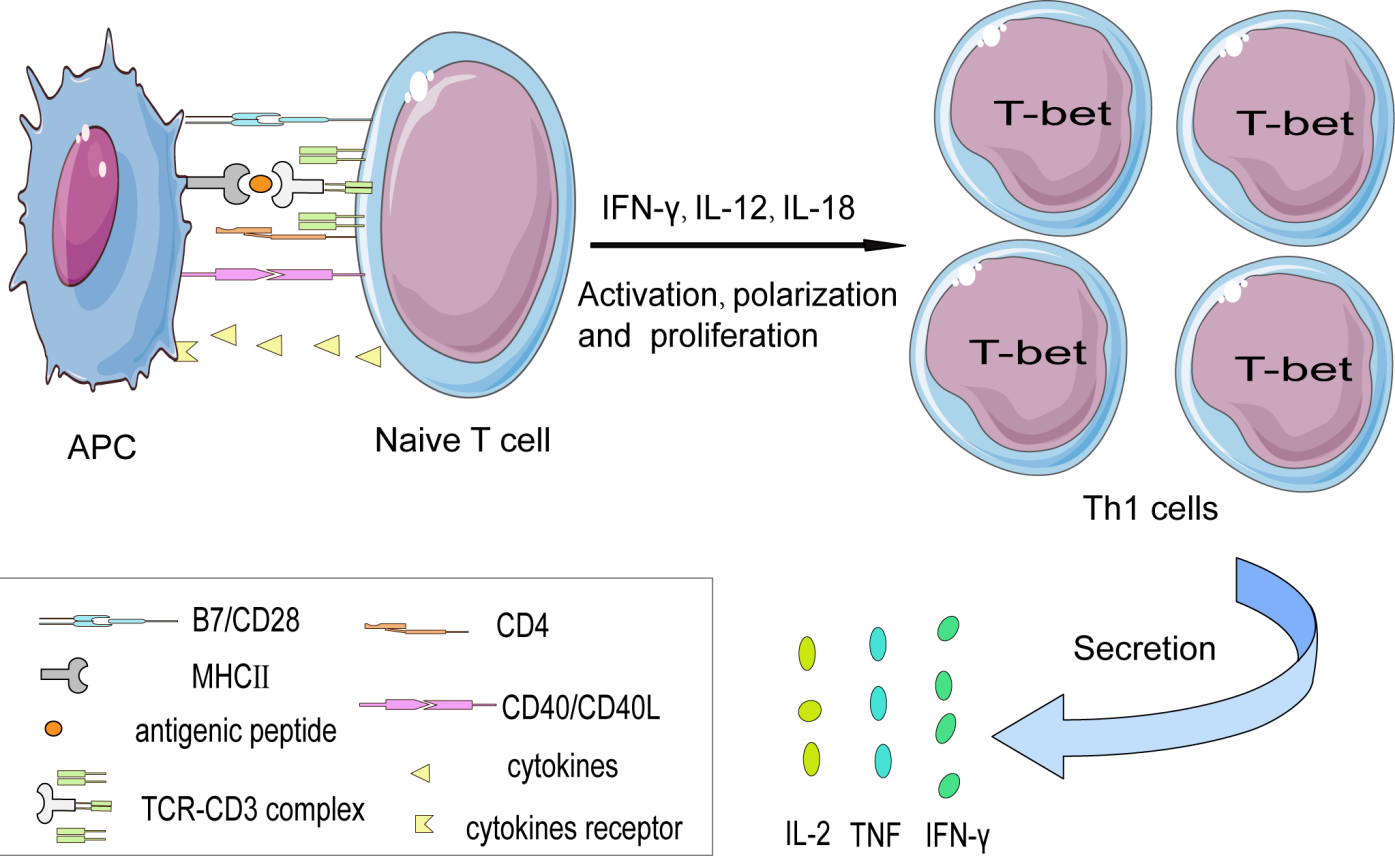
The interaction between naïve T cells and APCs in a cell contact manner. The activation of naïve CD4+ T cell subsets is partially dependent on the interaction between the TCR-CD3 complex and antigen peptide presented on MHC II protein by APCs. Besides, costimulatory signals such as the CD28-B7 family (CD80/CD86) and CD40L-CD40 axis are indispensable in promoting T cells’ full activation otherwise T cells might become anergy. Costimulatory molecules induce T cells to express the characteristic transcription factor— T-bet that favor their differentiation into the Th1 subset. And then secrete pro-inflammatory cytokines such as IFN-γ, TNF, and IL-2.

In addition, CD40-CD40L also exerts an indispensable role in T-cell activation. CD40 molecule expressed on APCs the activation and polarization of Th1 cells (34). Studies have shown that inhibiting of CD40L-CD40 signal transduction axis can significantly alleviate atherosclerosis. In atherosclerosis-susceptible mice, CD40L deficiency in CD4**^+^** T cells shows impaired Th1 polarization, mainly reflected in the reduced production of pro-inflammatory IFN-γ, thus alleviating the development of atherosclerosis (35). Furthermore, programmed cell death protein 1 (PD1) has been detected on T cells by CyTOF, indicating that under long-term chronic inflammation stimulation, T cells undergo terminal differentiation (16). In agreement with this, it has been reported that using PD1 monoclonal antibody (mAb) to treat end-stage tumors in patients is associated with an increased risk of atherosclerotic disease (36) (**Figure 2**).

### 2.3 Polarization of Th1 cells

When APCs activate naive CD4+ T cells, they can differentiate into different helper cell subsets (37). The characteristic differentiation of Th1 cells depends on the interaction of the co-stimulatory molecules expressed on the surface of both T cells and APCs. Besides, the milieu to which T cell is exposed amid the circulation and the plaque also affect their polarization (18). Previous studies have shown that the polarization of Th1 cells is mainly affected by IFN-γ, IL-12, and IL-18. On the contrary, IL-4, IL-10, and TGF-β can promote Th2 cell polarization while inhibiting Th1 cell differentiation.

#### 2.3.1 The characteristic transcription factor T-bet

The T-box protein T-bet, a characteristic transcription factor of Th1 cells, is not expressed in naive CD4+ T cells. It is believed to be an essential regulator of Th1 cell differentiation. Encoded by the TBX21 gene, it can directly bind to the IFN-γ DNA transcription sequence to activate and promote IFN-γ gene transcription to enhance the expression of IFN-γ. Besides, T-bet can promote the expression of the IL-12Rβ2 subunit to enhance the IL-12 signal pathway. Interestingly, studies have proved that increasing T-bet expression can re-transform the differentiated Th2 cells into Th1 cells (38).

IFN-γ, mainly produced by effector T cells, is capable of binding to its receptors on Th1 cells. IFN-γ exerts its effects by activating the intracellular molecular signaling network. The Janus kinase-signal transducer and activator of transcription (JAK-STAT) pathway is the best characterized one (39). Through the IFN-γ-JAK-STAT1-T-bet pathway, T-bet can promote the expression of IFN-γ, and IFN-γ secreted by Th1 cells can produce a positive feedback effect through the above pathway, thereby enhancing the Th1 phenotype (40).

In addition, IL-12 can stimulate the signal transducer and activator of transcription 4 (STAT4) in an IFN-γ independent manner, promoting the polarization of the Th1 cell. IL-12, mainly derived from cDC1s *in vivo*, acts with the IL-12 receptor on the Th1 cells’ surface and promotes the secretion of Th1 cells’ characteristic transcription factor IFN-γ through the IL-12R-STAT4-T-bet pathway. At the beginning of inflammatory diseases, IL-27, one member of the IL-12 cytokine family, can induce the expression of IL-12 receptors on the surface of naive CD4**^+^** T cells, thereby promoting IL-12-induced Th1 cell differentiation (41). During Th1 cell differentiation, the activated IL-12R signal pathway facilitates the expression of T-bet. STAT binding to TBX21-CNS-12 is critical for T-bet-induced polarization of naïve CD4**^+^** T cells into Th1 cells (42).

#### 2.3.2 Other transcription factors associated with Th1 polarization

T-bet interacts with Hlx, Runx, ETS-1, and Bhlhe40 to promote IFN-γ production and thus induce Th1 cells’ polarization (43–45). Other studies have shown that Bhlhe40 can regulate cytokine secretion in different helper T cell subsets, such as Th1, Th2, and Th17 cells, independent of subgroup restriction, and play a pro-inflammatory role by inducing GM-CSF secretion and inhibiting IL-10 secretion (46, 47). The transcription factor p73, a member of the p53 family, negatively regulates Th1 cell differentiation by binding to IFN-γ and IL-12Rβ2 genes (48). In addition to the traditional Th1 master regulator T-bet, other transcription factors have been found during Th1 cell differentiation, proliferation, and migration. For example, the runt-related transcription factors (Runx) family includes three family members, Runx1, Runx2, and Runx3. Each Runx family member performs unique functions at each stage, in which Runx3 works in conjunction with T-bet to directly inhibit IL-4 transcription. Recently, it has been demonstrated that other cytokines assist T-bet action (43).

The interaction among different transcription factors is involved in different cell lineage differentiation processes (49). For instance, T-bet inhibits the transcription of transcription factors such as GATA3 (the characteristic transcription factor of Th2) and ROR-γ (the characteristic transcription factor of Th17), thus inhibiting the naïve T cells polarization toward Th2 and Th17 during cell differentiation (50).

Thus, different T cell subsets differentiate amid a complex and delicate regulatory network. An important cross-regulation in the differentiation of CD4**^+^** T cells is inhibiting transcription factors essential for lineage determination. Recent studies have found that T-bet plays a key role in the differentiation and coordination of innate and adaptive immune T lymphocytes. T-bet can play different roles in the differentiation of different subsets through phosphorylation and modification of amino acids at different sites (42).

#### 2.3.3 The role of antigen-presenting cells in the polarization of Th1 cells

During differentiation of naive T cells into different Th cells *in vivo*, the role of antigen-presenting cells cannot be ignored. During Th1 cells polarization, antigenic peptides and MHCII molecules interacting with the TCR-CD3 complex significantly increase the T-bet expression (42). The most important APCs are dendritic cells (DCs). There are several different DCs subpopulations: conventional type 1 DCS (cDC1s), conventional type 2 DCS (cDC2s), plasmacytoid DCs (pDC), monocyte-derived DCS (moDC), and Langerhans cells (LC). Among the different classifications, cDCs are the most abundant and different in their ability to induce Th1 or Th2 responses. To promote Th1 cells polarization, it is necessary to induce the expression of cytokines, such as IL-12. The main source of IL-12 *in vivo* has been proved to come from cDC1s, which constitutively express IL-12β transcription and produce IL-12p40 protein, thereby enhancing Th1 differentiation (51). ILC is a kind of congenital lymphocyte lacking a specific antigen receptor. Recently, it was found that ILC plays a role in the polarization of Th cells. One way ILC affects the production of T cell responses is by secreting pro-Th1 or pro-Th2 cytokines. Nevertheless, there is still a lot to learn about mechanism, for example, how this mechanism compares to DC antigen presentation (40).

Studies have revealed that the saturated fatty acids, to which the T cell is exposed in the circulation, may have a pro-inflammatory influence on T cells, through activation and polarization of Th1 cells (52). Due to the plasticity of CD4**^+^** T cells, therefore, Th1 cells might derive not only from naive CD4**^+^** T cells but also from the trans-differentiation of mature differentiated effector T cells, including Th17, Treg, and Tfh cells (11).

## 3 Th1 cell homing to atherosclerotic lesions

T cells expressing CD3 molecules mainly exist in the fibrous cap of plaque and the arterial adventitia (also known as tertiary lymphoid organs) (53). However, it is not clear how they get there. The migration mechanism of lymphocytes to home to the adventitia remains unclear, including infiltrating the adventitia from the vascular lumen or through the vasa vasorum of blood vessels. In contrast, the healthy aortic wall is believed to be absent of microvessels (6, 54). Besides, Single-cell data from human plaques and peripheral blood showed that the number of Th1 cells in the plaques was higher than in the peripheral blood mononuclear cells.

Interestingly, it is long believed that the CD4**^+^** T lymphocytes activation occurs in secondary lymphoid organs (such as spleen and lymph nodes), and the expression of chemokine receptors, such as CCR5, CXCR3, and CXCR6, on the Th1 surface is significantly increased after activation. Th cells with an effector phenotype migrate into plaques by interacting with chemokines expressed by vascular endothelial cells, smooth muscle cells, and macrophages. Of note, it is also believed that naïve T cells can be primed directly in the vessel wall. The interactions between APCs and T‐cells in the arterial wall lead to the activation of local naïve T-cells and the production of pro‐inflammatory cytokines such as IFN-γ (55).

### 3.1 L-selectin and PSGL-1

L-selectin is a surface marker of naïve CD4**^+^** T cells. T cells that reside constitutively within the aortas are partially L-selectin dependent (56). One study has shown that L-selectin deficiency in wild-type mice can result in a 50% reduction in the lymphocyte number that homes to the artery, suggesting that L-selectin plays an important role in Th1 cell migration into the aorta. Besides, the loss of L-selectin significantly reduced the number of Th1 cells in the plaque area but did not alter the spatial localization of Th1 cells (56). Moreover, P-selectin glycoprotein ligand 1 (PSGL-1), the major leucocyte ligand for P-selectin, is key in promoting naïve T-cell recruitment to the aorta. A significant reduction of naïve CD4**^+^** T cells was identified in the anti-PSGL-1-treated aorta (19).

### 3.2 CCR5

The chemokine receptors can be divided into two main subfamilies according to the exact position of the two conserved cysteines in the N-terminal region: CC chemokines and CXC chemokines (57). C-C motif chemokines profoundly influence atherosclerotic plaque development. CCR5 usually binds to its ligands CCL3 and CCL5 to exert its function (58). It is reasonable to hypothesize that they are associated with Th1 cells homing to the plaques (38). Using flow cytometry, Campbell et al. demonstrated that the interaction between chemokines and their receptors expressed on plaque cells controlled T cell influx. CCR5 has been shown to regulate Th1 cells to infiltrate the inflammatory plaques. Notably, CCR5 is needed for CD4**^+^** T cells to infiltrate the atherosclerotic plaques. CCR5 gene knockout or CCR5 receptor blockage by specific antibodies can significantly alleviate atherosclerosis in murine models (59).

### 3.3 CXCR3

CXCR3, a member of the CXC motif family, is highly expressed in Th1 cells. CXCR3 has a crucial influence on Th1 recruitment into inflammatory tissues. The number of Th1 cells that influx into plaques depends on the high-level expression of CXCR3 induced by IFN-γ and its corresponding ligands CXCL9, CXCL10, and CXCL11 (60). Sc-RNA-seq research found a higher CXCR3 expression level in circulating CD4**^+^** T cells in symptomatic atherosclerosis patients compared to asymptomatic atherosclerosis patients, indicating that these cells can be activated and recruited into plaques to aggravate the disease (12).

### 3.4 CXCR6

CXCR6 is another chemokine receptor expressed on the Th1 cells’ surface. CXCR6 binds to CXCL16, mainly expressed on the plaques, thereby promoting the infiltration of activated Th1 cells into atherosclerotic lesions. In CXCR6^-/-^/ApoE^-/-^ mice, the atherosclerotic lesion size was significantly reduced, and the IFN-γ expression level was decreased greatly compared with ApoE^-/-^ mice (61). IL-2 can promote the expression of CXCR6 on Th1 cells and thus promote Th1 cells’ migration into the plaques, aggravating lesions. Therefore, CXCR6 is a key factor in atherosclerosis development (61). These results suggest that after activation in the second lymphoid organs, antigen-exposed T cells influx into the plaques through the interaction between chemokine receptors (CCR5, CXCR3, and CXCR6) expressed on their surface and their corresponding ligands expressed on plaques.

In addition to effector phenotype Th1, naïve T cells are not rare in plaques. However, it remains unclear where these aortic naive T cells originate. This raises new questions about the origin of Th1 cells in atherosclerotic plaques. Thus, it remains to be explored whether naïve T cells activation in the plaques is mediated by the interaction with APCs in the plaques or the secondary lymphoid organs and tissues and later infiltrates into the plaques.

## 4 The molecular mechanisms by which Th1 cells contribute to atherosclerosis

Accumulating evidence indicates that Th1 cells are implicated in the onset, development, progression, and rupture of atherosclerotic lesions. Th1 cells promote atherosclerosis development by secreting pro-inflammatory cytokines, such as IFN-γ, tumor necrosis factor (TNF-α), IL-2, IL-3, and lymphotoxin to induce chronic inflammation in the atherosclerotic lesions and facilitate foam cells formation (62), which might eventually cause dysfunction and erosion of endothelial cells integrity, resulting in thrombus formation and myocardial infarction or ischemic stroke.

IFN-γ is associated with the initiation and modulation of innate and adaptive immune responses, many of which have a pro-atherosclerotic role (63). It promotes the expression of adhesion molecules in endothelial cells to accelerate the recruitment and infiltration of Th1 into the sub-endothelium (64). IFN-γ directly influences the stability of the plaque by affecting macrophage polarization. It is the major factor associated with macrophage activation, inducing the expression of genes associated with lipid uptake and prime macrophages to produce pro-inflammatory chemokines and cytotoxic molecules such as TNF-α and IL-6 (65). Besides, IFN-γ can increase plaque vulnerability by accelerating macrophage and vascular cells apoptosis by inducing the release of matrix metalloproteinases (MMPs) (65). Thirdly, IFN-γ could inhibit vascular smooth muscle cell (VSMC) proliferation and extracellular matrix breakdown, weakening plaques more prone to rupture or erosion. IFN-γ could also inhibit the expression of collagen genes such as collagen I and III (64). One recent study revealed that IFN-γ could influence the metabolism of the endothelial cells in the coronary artery wall by impairing its glucose metabolism, resulting in a metabolic shift toward fatty acid oxidation, possibly harming endothelial cells’ function (66, 67).

In addition to IFN-γ, TNF-α is involved in the progression of atherosclerotic lesions. Although TNF-α is mainly synthesized by macrophages, Th1 cells also produce TNF-α. On the one hand, TNF-α can trigger and maintain local inflammatory responses by promoting pro-inflammatory cytokines secretion. TNF-α has two different receptors, TNFR1 (p55) and TNFR2 (p75) (68). Here we mainly discuss the role of the former because TNFR1 can activate the p38 mitogen-activated protein kinase (MAPK)/nuclear factor kappa-light-chain-enhancer of the activated B-cell (NF-kB) pathway, which plays a pro-inflammatory role by promoting pro-inflammatory factors, such as IL-1, IL-6, and GM-CSF (69). On the other hand, TNF-α can inhibit lipoprotein lipase and reduce the oxidative metabolism of fatty acids (68), thus leading to hypertriglyceridemia and stimulating the reactive oxygen species production, thus promoting atherosclerosis development (70). In humans, TNF-α promotes the penetration of circulating inflammatory cells into plaques by up-regulating adhesion molecules expressed on the surface of endothelial cells, such as vascular cell adhesion molecule 1 (VCAM-1) (71).

**Figure 3:**
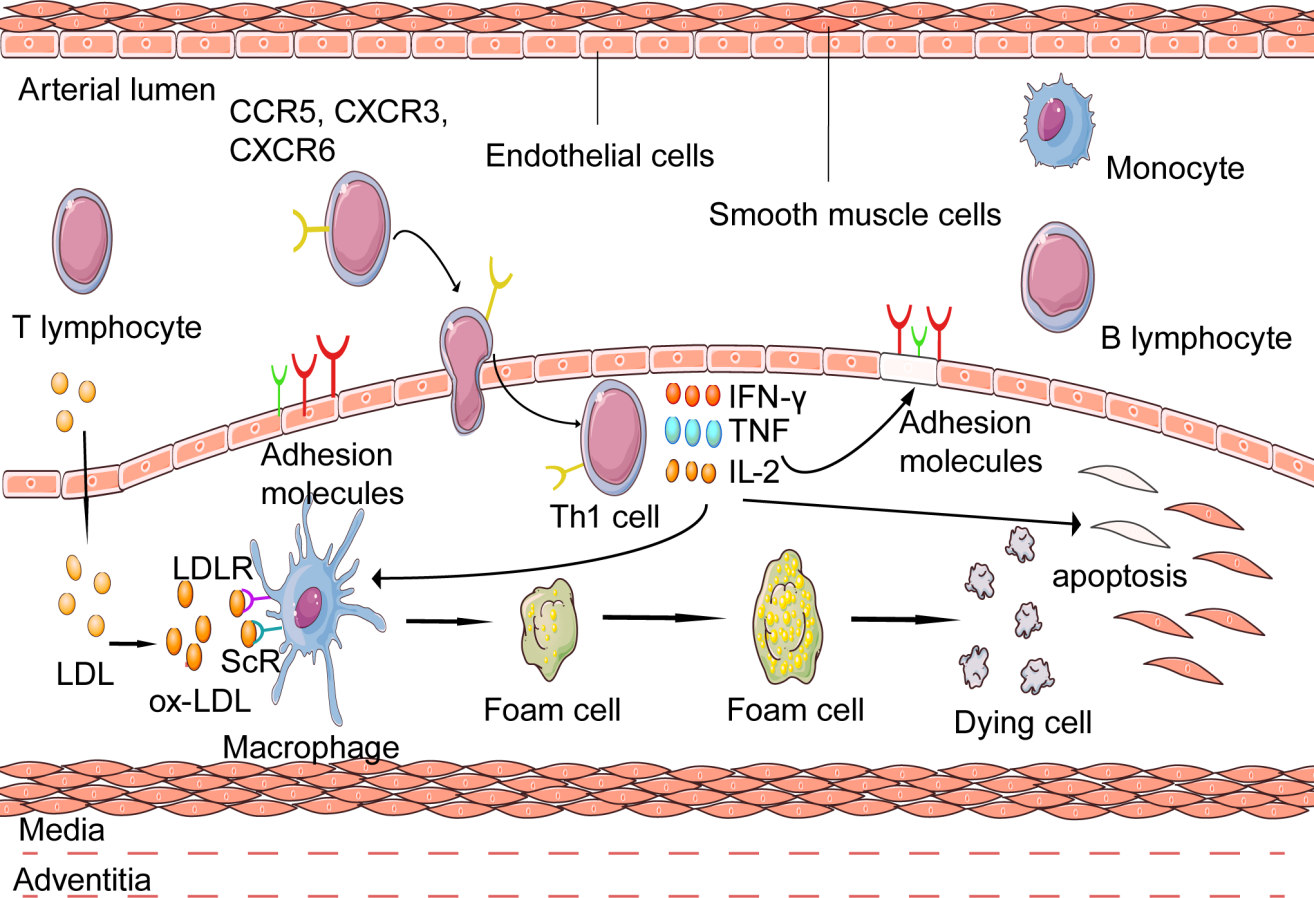
Activation of inflammatory response mediate by Th1 in atherosclerosis. Atherosclerotic disease is initiated when low-density lipoprotein (LDL) enters the arterial intima, undergoing various chemical modifications such as oxidation. Macrophages that accumulate ox-LDL become lipid-laden foam cells. Apolipoprotein B-100(ApoB-100)peptides are presented on MHCII molecular by antigen presenting cells(APCs) to activate naïve T cells. Then ApoB 100-reactive T cells become activated and proliferate under the influence of the milieu to which they are exposed. Naive T cells differentiate into Th1 cells and secrete pro-inflammatory cytokines such as IFN-γ, TNF, and IL-2. ApoB-100 reactive T cells are also found in atherosclerosis plaques, which activate macrophages and modulate the formation of foam cells.

The pro-inflammatory effects of the above IFN-γ and TNF-α have been verified in the experimental animal models. An experimental study showed that when ApoE^-/-^ mice were injected with exogenous recombinant IFN-γ, the plaque size decreased by 15% compared with the control group (72). While using ApoE^-/-^IFN-γ^-/-^ mice, their plaques size was smaller than those of ApoE knockout mice. Besides, the collagen content in the plaque fibrous cap was richer, indicating more stable plaques (72). In addition, the TNFR1 “knockout” mice had significantly reduced inflammation (71) (**Figure 3**).

## 5 Potential Th1 cell-based therapy

Atherosclerosis treatments mainly consist of lowering blood lipids, stabilizing plaque, and anti-platelet therapies. Statins, such as atorvastatin, mainly decrease the expression level of the co-stimulatory molecules such as CD83 and CD86, leading to a decreased capability of DCs to induce T‐cell activation and proliferation and Th1 differentiation (73). Traditional therapies might be effective at dampening the progression of atherosclerosis, but they are not capable of causing appreciable regression of existing plaques (74). Due to the pro-atherosclerosis role of Th1, therapeutic inhibition of Th1 cells is a novel and promising strategy to suppress atherosclerosis. However, no clinically available therapies target Th1 lymphocytes (74).

### 5.1 Vaccination

Influenza vaccines have already been used to reduce cardiovascular risk in older people (75), whereas the major mechanism remains unclear. Using vaccines to induce antibodies production to dampen the endogenous pro-atherogenic factors might become a more useful strategy but, at the same time, more challenging. Initially, the athero-protective effect of immunization with oxidized LDL (ox-LDL) was first observed in hypercholesterolemic rabbits (76). Then, several studies explored its effect in different atherosclerosis mouse models (77). However, when it comes to transferring the approach into clinical application, finding the antigen(s) responsible for activating protective immunity is a significant challenge. Studies have proved that the athero-protective mechanism of ox-LDL might activate Treg cells to exert a suppressive influence on Th1 cell response (78). Researchers have tried to find autoantigens from the entire LDL molecule to its core proteins, apolipoprotein subunits, such as ApoB-100 peptide. Apolipoprotein B100 (ApoB), the core protein of LDL, is the best candidate for an autoantigen that induces proliferation of pathogenic Th 1 cells (25).

In addition to the self-antigen, the adjuvants, including killed mycobacteria, mineral oil, and modified proteins, enhance the immune response, adding another challenge to advance the vaccine strategy into future clinical applications. In mouse studies, the most commonly used adjuvant is the Freund adjuvant. However, it tends to selectively promote Th1 responses, limiting its use in future clinical use (79). To solve this problem, Kuang-Yuh and his group used P210, a ApoB-100–related peptide and self-assembling peptide amphiphile micelles (P210-PAMs) to reduce the atherosclerosis development in ApoE*^–/–^* mice. Interestingly, the outcome aligns with the P210-PAM as a potential vaccine candidate to treat human atherosclerosis-associated disease (80).

In addition to active immunization, vaccines have also been used to induce immune tolerance. Administering antigens through the mucosal route is another attractive way to induce self-antigens tolerance, which has been proven to reduce atherosclerosis (81). This strategy mainly activates the Treg, located in the mucosal lining of airways and intestines, to exert a protective effect on atherosclerosis. Treg cells represent another subset of CD4**^+^** T helper cells regulating inflammation through the secretion of cytokines such as IL-10 and TGF-β (82). Evidence suggests that the number of Treg cells decreases during atherosclerosis development. Single-cell RNA sequencing has revealed a new subset, the Th1/Treg subset, characterized by T-bet and Foxp3 (characteristic Treg transcription factor) positivity (9), suggesting that during atherosclerosis progression, Treg cells lose their protective properties and develop into Th1 cells with atherogenic effects, further demonstrating the Th1 cell phenotypes’ plasticity during atherosclerosis progression (16). Altogether, these conclusions confirm that more attention should be placed on the strategies to maintain the functioning Treg population. In mature Tregs, the activation of glucocorticoid-induced tumor necrosis factor receptor-related protein (GITR) enhances proliferation; therefore, it might be a new target in the background of regulatory T cells (83). Another way is to induce the production of neutralizing antibodies, such as targeting PCSK9 or cholesterol lipid transfer protein, thereby playing a protective role in atherosclerosis (84). However, the concept of vaccine use against atherosclerosis was proposed for more than 20 years. In addition, preclinical trials have provided some positive results, but the chances of translating these experimental results into clinical trials have been low. Furthermore, it remains unconfirmed whether the vaccines can play a protective role after the initiation as well as the formation of atherosclerotic plaque.

### 5.2 Blockade the cytokines associated with Th1 cells

#### 5.2.1 IL-12

IL-12 is an effective cytokine in promoting naïve T cells differentiation into Th1 cells. Strong evidence shows the effectiveness of IL-12-specific antibodies as an atherosclerosis treatment. Specific IL-12-blocking antibodies have been used in clinical trials to treat psoriasis (85). However, further studies on these antibodies are needed to understand how IL-12 blockade can influence cardiovascular disease development. It remains unclear whether IL-12-specific antibodies can reduce Th1 cells’ differentiation and alleviate their atherosclerotic effect in humans. IL-12 and IL-35 belong to the IL-12 cytokine superfamily and share the same subunit, p35. IL-35 plays a vital role in maintaining Treg-mediated immune homeostasis and is considered a functional cytokine of Treg (86). IL-35 may maintain protective function of Treg against hyperlipidemia by increasing CCR5 amplification mechanism (87). It is believed that Treg has an anti-atherosclerosis effect. However, it is unclear whether blocking IL-12 by a specific antibody affect the protective effect of IL-35. The effect of IL-12α subunit p35 (IL-12p35) on atherosclerosis has been studied by knocking out the IL-12p35 gene. The results showed that IL-12α subunit deficiency decreased the infiltration of CD4+T cells and macrophages in atherosclerotic lesions and increased the content of vascular smooth muscle cells and collagen in ApoE^-/-^ mice, suggesting that IL-12p35 deficiency effectively alleviates atherosclerosis and promotes vascular smooth muscle cells development (86).

#### 5.2.2 IFN-γ and TNF-α

There is evidence supporting the pro‐atherogenic role of IFN-γ and TNF‐α. TNF‐α, when bound to its receptors, activates NF‐κB and p38 MAPK inducing the transcription of pro-inflammatory genes such as IL-8 in lymphocytes. Specific antibodies were used to block IFN-γ and TNF‐α binding to their receptors (88). There are examples of treating autoimmune diseases such as rheumatoid arthritis (RA) and Crohn’s disease with a mAb against IFN-γ. Clinical trials also use TNF-α-associated mAbs to treat vitiligo (89). However, it is unavoidable that the mAbs may reduce the anti-infection ability of the immune system and increase the risk of suffering opportunistic clinical infections.

### 5.3 Blockade other receptors expressed by Th1 cells

#### 5.3.1 CCR5

CCR5, a chemokine receptor, is also a co-receptor for human immunodeficiency virus (HIV) to invade CD4**^+^** T cells (58). CCR5 is expressed on the Th1 cell surface and mediates their infiltration into atherosclerotic plaques. CCR5-specific antibodies have been used to treat HIV infection (7). The CCR5 antagonist Maraviroc is approved in American and European markets with an established long-term safety. Maraviroc decreases atherosclerosis progression in atherosclerosis-prone mice and HIV-positive individuals (90). Hence, exploring this drug as a candidate for treating atherosclerosis seems advisable (91).

#### 5.3.2 EGFR

The epidermal growth factor receptor (EGFR), a receptor bound to the cell membrane, has been well-researched during lung cancer development. Several EGFR inhibitors, such as erlotinib and cetuximab, have been successfully developed as cancer treatments. Researchers have recently found that leukocytes, especially Th1 cells, also express EGFR (92). EGFR inhibitors can induce T cell anergy *in vivo* and *in vitro* and repress atherosclerosis development. Hence, EGFR may be another novel target to block to combat atherosclerosis (93) (**Table 1**).

**Table 1:** Summary of key drugs associated with Th1 cells to treat atherosclerosis.

Target	Mechanism	Intervention	Clinical application	Reference
IL-12	IL-12 receptor antagonist	ustekinumab	Psoriasis	(85)
TNF-α	TNF-α monoclonal antibody	adalimumab	vitiligo	(94)
		infliximab		(89)
IFN-γ	IFN-γ monoclonal antibody	Fontolizumab	Crohn’s disease and rheumatoid arthritis	(88)
EGFR	EGFR tyrosine kinase inhibitors	AG-1478、erlotinib、cetuximab	lung cancer	(93)
CCR5	CCR5 receptor antagonist	Maraviroc	HIV	(90, 95, 96)
		TAK779		(97)

## 6 Conclusions and future perspectives

Numerous pieces of evidence indicate that atherosclerosis is a chronic progressive inflammatory disease involving the innate and adaptive immune systems. Specifically, T lymphocytes, part of the adaptive immune system, comprise over 30% of the leukocytes present in atherosclerotic plaques of mice and humans. Significantly, the Th1 cell, expressing the transcription factor T-bet, has been proven to play a significant role in the development and progression of atherosclerotic plaques. Evidence shows that Th1 cells have atherogenic effects while Treg cells have anti-atherogenic properties.

However, the antigenic specificity of T cells remains unclear, although LDL and ApoB100 may be the most likely candidates to cause atherosclerosis. Moreover, identifying the specific antigens to activate T cells *in vitro* has been challenging. Besides, the precise mechanism and location leading to T-cell activation, proliferation, and migration remain further explored.

Although secreting pro-inflammatory cytokines such as IFN-γ confirmed that Th1 cells are pro-atherosclerotic, no clinically applicable therapies for atherosclerosis related to Th1 cells are available. Moreover, whether the immune factor of atherosclerosis can be addressed by anti-inflammatory therapies targeting cytokines, chemokines, and other inflammatory pathways remain unknown. Vaccination may be a promising candidate as a atherosclerosis mediator. Several vaccines are proven effective in animal models, but the translation of these approaches into clinical applications is still facing great challenges. Based on the previous discussion, therapeutic approaches targeting Th1 cells remain under exploration. Nevertheless, it is urgent o explore innovative therapeutic approaches for atherosclerosis.

## Author contributions

Elaborated the figures and wrote the manuscript: JC and XX. Reviewed topics and concepts: LN, XG, FZ, CW and YX. Conceived, reviewed and discussed concepts in the manuscript: LM. All authors contributed to the article and approved the submitted version.
